# Simplified vacuum dressing system: effectiveness and safety in wounds management

**DOI:** 10.1590/acb370906

**Published:** 2022-12-12

**Authors:** Sandro Cilindro de Souza, Carlos Maurício Cardeal Mendes, José Valber Lima Meneses, Rosana Menezes Dias

**Affiliations:** 1 Ph.D. Universidade Federal da Bahia – Instituto de Ciências de Saúde – Programa de Pós-Graduação – Salvador (BA), Brazil.; 2 Ph.D. Universidade Federal da Bahia – Faculdade de Medicina – Departamento de Cirurgia Plástica – Salvador (BA), Brazil.; 3 Nurse. Secretaria da Saúde do Estado da Bahia – Hospital Carvalho Luz – Salvador (BA), Brazil.

**Keywords:** Wounds and Injuries, Occlusive Dressings, Negative-Pressure Wound Therapy, Plastic Surgery

## Abstract

**Purpose:**

Negative pressure wound therapy (NPWT) has revolutionized wound care, but its high cost reduces the procedure’s availability. To solve the problem, streamlined vacuum dressings systems have been proposed, but the utility of these devices has been poorly studied. The objective of this study was to evaluate a simplified vacuum dressing system model (SVDM).

**Methods:**

Randomized clinical trial in which wounds were treated with SVDM compared to a complex occlusive dressing (silver hydrofiber, SHF). The analyzed outcomes were cleaning, presence of granulation tissue, clinical appearance, and indication for surgical closure of wounds.

**Results:**

Fifty injuries were treated (25 in each group), most located on lower limbs. SVDM proved to be more effective than SHF in the evaluated outcomes. Wound recalcitrance reduced the effectiveness of the equipment used. Despite its efficacy, complications occurred, the most frequent related to dressing changes: minor bleeding, foam adherence to a wound bed, and pain. Only for bleeding no favorable risk-benefit ratio was found. There were no severe complications, worsening conditions of injuries, or deaths.

**Conclusions:**

SVDM proved to be an effective and acceptably safe device for managing studied wounds.

## Introduction

Since its introduction two decades ago^1^^,^ 2, the benefits of negative pressure wound therapy (NPWT) have revolutionized wound management in several medical specialties, including angiology, gynecology, orthopedics, pediatrics, and plastic surgery^3^^–^ 10. However, the high technology makes the therapy costly and reduces its use in low-resource institutions^11^^,^ 12. To solve the problem, simplified vacuum dressings (SVD) systems have been proposed^11^^,^ 13 ^–^ 15. However, these devices have been criticized due to the use of rudimentary materials, difficulty in sealing wounds, and the impossibility of controlling subatmospheric pressures^3^^,^ 11 ^,^ 16 ^–^ 18.

Even when the cost is not an issue, the best treatment may be challenging to obtain or not be available, so knowledge of effective second indication treatments becomes essential^19^. Surgical treatment, being invasive, may result in complications and scarring (for example, graft donor areas). Thus, methods of promoting spontaneous healing (such as SVD) and avoiding surgical procedures are advantageous for patients and medical teams^4^.

Most of the studies available on SVD do not use comparison groups and present limited methodologies^11^^–^ 13 ^,^ 15 ^,^ 16 ^,^ 20. Considering the low frequency of clinical trials in surgical specialties^21^, conducting these trials becomes vital to obtain more evidence on the effectiveness of SVD.

The objective of this research was to evaluate the usefulness of an SVD model (SVDM) through the analysis of its efficacy and safety in wound management.

## Methods

Randomized superiority clinical trial, blinded and with two parallel arms, carried out from January 1, 2017, to May 1, 2020, at Roberto Santos Hospital (RSH), the largest public hospital in the state of Bahia (Brazil), with 640 beds. It is a highly complex teaching hospital and reference for multiple specialties related to the management of acute and chronic wounds, including traumatology, gastrointestinal surgery, neurosurgery, pediatric surgery, vascular surgery and gynecology.

The study was registered with the Brazilian Registry of Clinical Trials (RBR-5c8y6v) and followed the CONSORT 2010 recommendations^22^. The study was approved by the RSH Research Ethics Council (CAAE 55556816.7.0000.5028) and carried out following the Declaration of Helsinki. A Free and Informed Consent Form was obtained from participating patients.

A sample of 50 patients was calculated using the R statistical software (www.r-project.org), assuming a mean expected success rate of 98% for the SVDM group and 72% for the control group, with a margin of superiority of 25%. A test power of 80% and a significance level of 5% were assumed. Patients were sequentially admitted into the treatment (SVDM) and control groups (hydrofiber silver [SHF], Aquacel Ag+ Extra, Convatec Inc., ER Squibb & Sons, North Caroline, USA) following a list of random numbers generated in statistical software R. The statistical analysis used was by treatment protocol.

Adult patients hospitalized for acute (< 3 months) or chronic (≥ 3 months) wounds were included in the study. Subjects with decompensated systemic disorders (cardiac, thyroid, renal, pulmonary, hepatic, arterial hypertension, severe anemia, severe malnutrition, and coagulopathies) were not included. Painful wounds, infected wounds, wounds associated with perilesional dermatoses, allergic reactions, malignant neoplasms, and exposure to underlying exposed vessels, nerves, or viscera were also not included. The emergence of serious complications (e.g., hemorrhage, allergic reactions, sepsis, extensive necrosis, severe pain), decompensation of previously controlled systemic disorders, and deaths not attributable to the dressings were exclusion criteria used.

**Figure 1:**
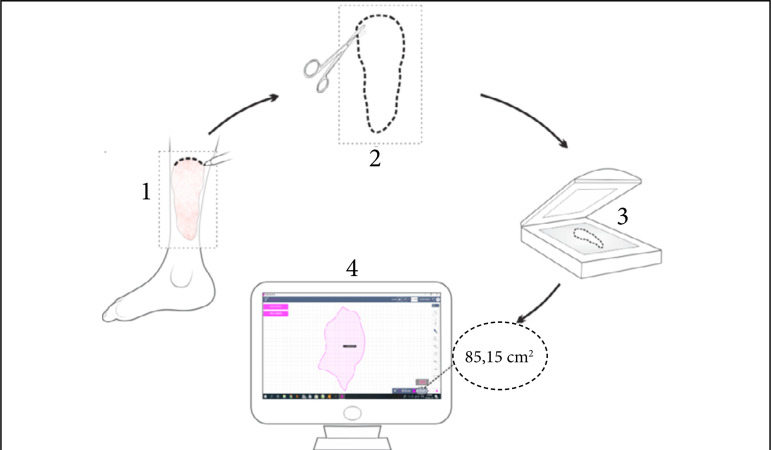
Measurement of wounded areas. (1) Decal of lesion contours using transparent acetate film; (2) Clipping of the demarcated area, obtaining a two-dimensional pattern (template); (3) Digitalization; (4) Computerized measurement of the injured area.

**Figure 2:**
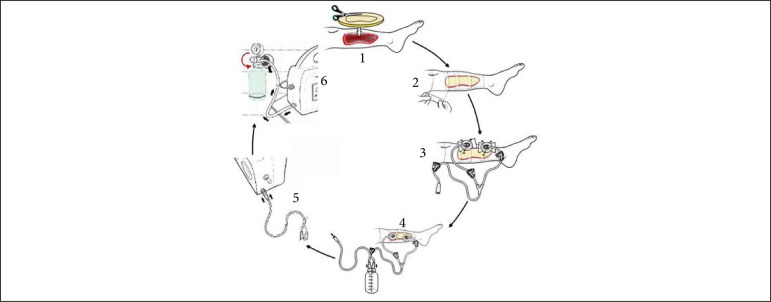
Applying the SVDM. (1) Cutting and placing the foam on the lesion; (2) Sealing the foam using a transparent polyurethane adhesive film; (3) Placing suction cups on one or two holes (2 cm) made in the film on the foam; (4) Suction tube connection to the liquid collection canister; (5) Connection of the canister to the control unit; (6) Activation of the SVDM and adjustment of the subatmospheric pressure.

**Figure 3:**
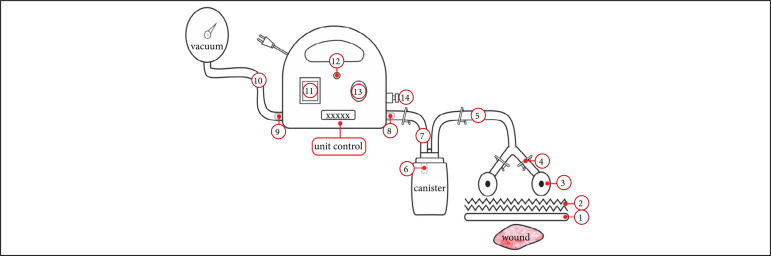
SVDM setup. (1) Foam; (2) Adhesive film (polyurethane); (3) Suction cup; (4) Clip cuts flow; (5) Drainage tube; (6) Filter; (7 and 10) Connecting tubes; (8) Air inlet; (9) Air outlet; (11) Timer display (digital); (12) Start button; (13) Vacuum gauge display (analog); (14) Vacuum adjustment knob.

Wounded areas were obtained using the SketchandCalc application (www.sketchandcalc.com, Fig. 1). Application and SVDM are shown in Figs. 2 and 3. SVDM was regulated with a pressure of –125 mmHg. The first dressing was used in continuous mode and the others in intermittent mode (5 min of vacuum and 2 min without vacuum)^2^^,^ 23. In both groups, debridement was performed to remove devitalized tissue occasionally present. Changes were made at ≥ 50% saturation of dressings to avoid unpleasant odor^24^. Patients were followed for 14 days or until the granular lesion (≥ 75% of the raw bed covered by healthy-looking granulation tissue).

The effectiveness of SVDM to improve lesions was evaluated by developing granulation tissue with a satisfactory appearance (*granulation* outcome) and the removal of dirt residues and debris that covered injured surfaces (*cleaning* outcome). Wounds were classified as clean or granulated only if more than 75% of their surface were clean or covered by healthy-looking granulation tissue. The power of SVDM to improve the quality of wounds (*clinical* outcome) and the indication of closure by surgical procedures (*surgical* outcome) was also evaluated. Before and after treatments, outcome analysis was performed using digital pictures (Sony W830 Silver, 20.1 megapixels). The evaluation was performed blindly by two plastic surgeons calibrated by observing 50 photographs of wounds unrelated to the clinical trial. Kendall’s W coefficient was used to determine inter-rater agreement (results: W: 0.5 to 1.0: substantial to excellent, according to Landis and Koch criteria)^25^.

Statistically, granulation and cleanliness were the study-dependent variables as they were the observed efficacy outcomes. Results were classified as unsatisfactory (grades 1 to 3: absent to good) or satisfactory (grade 4: excellent). Type of dressing (and SHF) was the primary independent variable (intervention) and variables sex, age (categorized as ranges [50.0–85.1 and 14.9–50.0]), diabetes, body mass, arterial hypertension, other comorbidities, and acute or chronic wound were covariates submitted to statistical modeling (Poisson regression) to obtain measures of association (relative risk [RR]; absolute risk rise; number needed to treat [NNT]; relative risk rise: direct measurement of efficacy^26^) considering the imbalance of variables between groups after randomization. The safety of SVDM was analyzed through the incidence of complications and quantification of the risk-benefit ratio (efficacy adjusted for adverse effects). The study assumed an overall ? error of 0.05.

## Results

**Figure 4:**
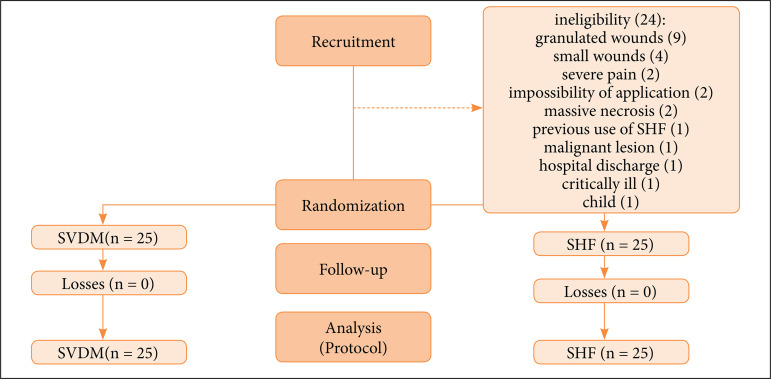
Flow diagram.

Of 74 inpatients evaluated, 24 were not included because they did not meet the inclusion criteria (Fig. 4). Patients studied were mainly men (SVDM: 52% vs. SHF: 68%), mestizos (SVDM: 72% vs. SHF: 84%), nonobese (88%, both groups), and mean age in the age group 6^th^ decade (SVDM: 55 years vs. SHF: 50 years, Table 1).

**Table 1:** Demographic characterization of samples according to groups.

Variable	SVDM (n = 25)			SHF (n = 25)	
	Mean (SD) (CV%)	Min/Max		Mean (SD) (CV%)	Min/Max
Age (years)	55 (14) (25)	29/85		50 (16) (32)	15/79
Weight (kg)	67 (16) (23.9)	47/108		68 (15) (21.8)	43/103
Heigh (cm)	164 (11) (6.9)	145/184		166 (12) (6.9)	154/180
	**n**	**%**		**n**	**%**
**Sex**					
Men	13	52		17	68
Women	12	48		8	32
**Ethnicity**					
Brown	18	72		21	84
Black	5	20		2	8
White	2	8		2	8
**BMI**					
Low weight	2	8		3	12
Normal	10	40		11	44
Over weight	10	40		8	32
Obesity	3	12		3	12

SD: standard deviation, CV%: coefficient of variation (percentage), BMI: body mass index (kg/cm^2^).

The wounds showed moderate areas (SVDM: 133 cm^2^ vs. SHF: 102 cm^2^), with no statistically significant differences between groups (Sd = 0,01; p = 0,46; Table 2). The preferred location was the lower limbs (SVDM: 64% vs. SHF: 72%). The etiology was predominantly postsurgical (28%, both groups). Treated wounds showed more frequently up to 30 days of evolution (SVDM: 76% vs. SHF: 64%). Recalcitrant wounds predominated in both groups (SVDM: 84% vs. SHF: 76%; Table 3). SAH (SVDM: 32%, SHF: 48%) and DM (SVDM: 28%, SHF: 48%) were the comorbidities most associated with injuries (Table 4).

**Table 2:** Wound areas (cm^2^).

SVDM (n = 25)			SHF (n = 25)		Sd	p
Md(IQR)(CVMd%)	Min/Max		Md(IQR)(CVMd%)	Min/Max		
133(103)(77)	30/279		102(140)(138)	24/391	0.01	0.464

Md(IQR): median and interquartile range; CVMd%: coefficient of variation (median, in %); Sd (standardized difference): measure of association constructed from the Mann–Whitney U statistic: absent: 0.0–0.2; small: 0.2–0.5; moderate: 0.5–0.8; large: >0.8.

**Table 3:** Location, type, and evolution of wounds according to groups.

Variable	SVDM (n = 25)			SHF (n =25)	
	n	%		n	%
**Body part**					
Head/neck	0	0		0	0
Trunk	7	28		7	28
Limbs	18	72		18	72
**Types**					
Postsurgical	7	28		7	28
Trauma	6	24		4	16
Infection	6	24		5	20
Bite	2	8		1	4
Pressure sore	2	8		3	12
Burn	1	4		2	8
Venous ulcer	1	4		2	8
Myiasis	-	-		1	4
**Evolution**					
Acute	15	60		12	48
Chronicle	10	40		13	52
Recalcitrant	21	84		19	76

**Table 4:** Distribution of comorbidities according to groups.

Comorbidity	SVDM (n = 25)			SHF (n =25)	
	n	%		n	%
SAH	8	32		12	48
DM	7	28		12	48
Smoking	2	8		5	20
Obesity	3	12		3	12
Alcoholism	3	12		6	24
Others	6	24		7	8

SAH: systemic arterial hypertension, DM: diabetes mellitus.

The efficacy of SVDM in comparison to SHF after using the Poisson regression was 169% for granulation and 151% for cleaning, and it is necessary to treat 1 to 2 patients to be successful in these outcomes (p = 0.0001 for granulation and p < 0.0001 for cleaning). The recalcitrance of wounds had a reducing effect on the effectiveness of the SVDM on granulation and cleaning (respectively, RR = 0.57, p = 0.0037; RR = 0.62, p = 0.0108). In addition, when taking recalcitrance into account, a bias in the gross RR is reduced (granulation: 5.08%, cleanliness: 4.60%). For clinical and surgical outcomes, the effectiveness of the SVDM on the effect of SHF was 157%, and it is necessary to treat 2 to 3 patients to achieve success for both outcomes (p = 0.0061). There was a greater probability of success in tissue granulation and cleaning lesions in the SVDM group with the nonrecalcitrant wound (granulation: 59.1%; cleaning: 58.5%). In other words, even with the decrease in the effectiveness of the SVDM for recalcitrant wounds, the device still proved to be more effective than the SHF for granulation and wound cleaning. In contrast, the lowest probability of success was found in recalcitrant wounds treated with hydrofiber (granulation: 23.3%; cleanliness: 25.9%; Table 5).

**Table 5:** Probability of success (obtained from the Poisson model) in granulation and wound cleaning according to the combination of dressings and wound recalcitrance.

Dressing	Recalcitrant wound	Probability (%)	
		Granulation	Cleaning
SVDM	No	59.1	58.5
	Yes	45.1	46.7
SHF	No	34.9	35.0
	Yes	23.3	25.9

Table 6 presents the chance of benefit (likelihood of being helped or harmed [LHH])^26^ of SVDM considering efficacy and adverse effects. The most found adverse effects in the SVDM group were bleeding, foam adhesion to the wound, and pain. As the SVDM NNT for granulation was 1.6, it is expected that for every 160 patients treated with SVDM, 100 granulated injuries, 109 bleeding patients, and 6 patients with hematoma (or necrosis) will be obtained. Only for bleeding, the expected benefit with the SVDM did not outweigh the harm (LHH = 1). For other adverse effects, the benefits outweigh the complications. For the wound cleaning outcome, for every 150 patients treated with SVDM, it is expected to obtain 100 clean wounds, 102 bleeding patients, and 6 cases of hematoma; here, the benefit of using the SVDM is expected to be greater than the losses caused by each complication (LHH > 1). Finally, it will be necessary to treat 230 individuals with SVDM to expect to obtain 100 wounds with a satisfactory clinical appearance (or 100 injuries suitable for early closure through surgery), 156 bleedings, and 9 cases of hematoma; in contrast to previous outcomes, bleeding was the adverse effect whose presence outweighed the benefit of treatment with SVDM (LHH < 1). However, bleeding, in addition to being the most frequent negative effect in SVDM (68%) and having a number needed to harm [NNH]^26^ of 1.7 (for approximately 2 patients treated with SVDM, 1 is expected to have bleeding), when considering the four outcomes studied, it was responsible for more harm than help (LHH = 0.7) or matched the benefit (LHH = 1.0), or even contributed to there being little benefit (LHH = 1.1). In summary, SVDM proved to be statistically capable of producing more benefits for most complications (LHH: 1.1 to 16.7), except for bleeding and dressing adherence (respectively, LHH: 0.7 and 0.8) in the clinical and surgical outcomes. SVDM group had nine times higher pain levels (visual analog scale: SVDM: 415 vs. SHF: 45).

**Table 6:** Efficacy vs. adverse effect for SVDM in granulation, cleaning, clinical evaluation, and indication for surgical wound closure: chance of benefit or harm (LHH).

Complication	Incidence (%)	Expected n^o^ of complications taking NNT into account	NNH complication	LHH
**In wound granulation (NNT = 1.6)**				
Bleeding	68	1.09	1.7	1.0
Dressing adherence	60	0.96	1.9	1.2
Pain	52	0.83	2.5	1.6
Maceration	24	0.38	5.0	3.1
Contact dermatitis	8	0.13	12.5	7.8
Necrosis or hematoma	4	0.06	25.0	15.6
**In wound cleaning (NNT = 1.5)**				
Bleeding	68	1.02	1.7	1.1
Dressing adherence	60	0.90	1.9	1.3
Pain	52	0.78	2.5	1.7
Maceration	24	0.36	5.0	3.3
Contact dermatitis	8	0.12	12.5	8.3
Necrosis or hematoma	4	0.06	25.0	16.7
**In clinical evaluation or indication for surgical wound closure (NNT = 2.3)**				
Bleeding	68	1.56	1.7	0.7
Dressing adherence	60	1.38	1.9	0.8
Pain	52	1.18	2.5	1.1
Maceration	24	0.55	5.0	2.2
Contact dermatitis	8	0.18	12.5	5.4
Necrosis or hematoma	4	0.09	25	10.9

NNT: Number needed to treat; NNH: Number needed to harm; LHH: Likelihood of being helped or harmed = (1/NNT)/(1/NNH): LHH > 1: The patient has more benefits than the risk of complications; LHH < 1: There are more harms than benefits; LHH = 1: The benefits equal equal harms^26^.

## Discussion

In the present study, improvement in wound cleaning was not considered a single enough outcome to determine the effectiveness of the SVDM, as lesions need to be clean and satisfactorily covered by granulation tissue to allow healing^27^. The effectiveness of SVDM in improving the appearance of wounds (clinical outcome) and the indication of closure by surgical procedures (surgical outcome) was additionally evaluated. In clinical practice, the last two evaluations are more useful, as they allow classifying injuries as satisfactory or unsatisfactory and, thus, if they can be resolved earlier through surgery. In all analyzed outcomes, the superiority of the vacuum dressing was found, with an efficacy for cleaning the wound of 151.0% (p < 0.0001), 169.0% (p = 0.0001) for granulation and of 157.0% (p = 0.0061) for clinical and surgical evaluations in relation to SHF.

Results obtained indicate that SVDM improved the factors considered essential for the treatment of wounds (cleaning and granulation), optimized the clinical appearance, and increased the indication for surgical closure to injuries. This latter outcome is vital for surgeons as it corresponds to the minimum necessary time interval in which wounds need to be treated with dressings before they become suitable for therapeutic closure. It reflects the *bridge concept* in which the NPWT is used as an effective means between the first handling of the wound bed until its definitive coverage. In this spectrum, NPWT acts as a method of simplifying surgical procedures by optimizing wounds to allow, for example, closure by direct sutures or grafts instead of complex flaps^28^. In the current clinical trial, the coincidence of results between clinical and surgical outcomes also suggests that they are associated, that is, that the indication for surgical closure depends on how the wounds are considered by direct observation.

Quantitative evaluations of NPWT effectiveness are limited; those available use poor methodologies based on self-opinion, on outcomes without definition, and often impossible to be fully understood^5^^,^ 29. For example, satisfactory results with standard vacuum dressings were described in an uncontrolled paper as being for 95% of patients, but based on the authors’ judgment and without identifying any evaluation criteria^30^. Despite presenting these same methodological deficiencies, satisfactory results associated with the use of SVD have been similar to those obtained with the present study, varying in the researched literature between 74.2% to 100%^11^^,^ 14 ^,^ 15 ^,^ 20.

Regarding the development of granulation tissue, only two randomized trials were found (vacuum assisted closure system [VAC] vs. wet gauze): VAC: increase in the granulated surface: 61.1%, p = 0.001^31^, and increase in granulation volume: VAC: 63% to 104%, p = 0.01^2^. In addition, in a comparative study, an SVD model was associated, on average, in 10 days of treatment, with a greater cover of the raw surface by granulation tissue (SVD: 71.4% vs. wet gauze: 52.9%, p = 0.000082)^14^. NPWT foams have been associated with profuse^32^^–^ 34 and rapid granulation tissue development^14^^,^ 35, possibly related to greater intensity of microdeformations^36^. Such millimeter protrusions deform cells contained in them, which stimulates cell proliferation and results in the development of granulation tissue^4^^,^ 36. In the use of SVDM, microdeformations were observed as papules (1 mm) produced by penetrating the injured surface into pores of the foam with the application of suction. Microdeformations were not observed with SHF, with the hydrofiber being associated with smooth, pale granulation tissue development. The more quality cleaning associated with SVDM was attributed to the continuous drainage of exudates and foam removal, resulting in an avulsion of debris that penetrated the material’s pores^2^^,^ 32 ^,^ 36. Other randomized trials have also associated NPWT with earlier development of granulation tissue^35^, with differentiated results from vacuotherapy being observable since the first dressing change^37^.

NPWT is indicated for the treatment of recalcitrant wounds of various etiologies^35^. In the present study, recalcitrance represented a therapeutic challenge because, in addition to presenting a high occurrence (SVDM: 84%, n = 21), it reduced the effectiveness of SVDM to granulate and cleanse lesions (respectively, RR = 0.57, p = 0.0037; RR = 0.62, p = 0.0096). The decrease in efficacy in wound cleaning was, in general, supposedly due to the presence of recalcitrant wounds, which are acute or chronic injuries that do not respond expectedly (slow or absent improvement or worsening of the wound) to antisepsis, washing, and use of conventional or complex occlusive dressings in terms of cleaning, development of granulation tissue and healing^38^.

Complications most frequently found in the SVDM group occurred when dressings changed: bleedings with foams removal, adherences of the foams to the wound bed, making it difficult to remove, and pain during foam removal. Bleedings, in addition to occurring in most SVDM (2/3), were also the most unable concerning other complications since SVDM did not show a very favorable risk-benefit just about this problem (LHH ≤ 1.1). However, all bleeding was mild and self-limited. On the other hand, despite the good risk-befit ratio, adherence foam and pain were not so soft because they resulted in bleedings, longer exchange time of dressings (in case of adherence), and direct suffering from patients (in case of pain), although of short duration. The present study has found that adherence is a disadvantage associated with vacuotherapy. It can also result in infection due to the retention of foam fragments^11^.

The pain was more intense in the SVDM group and reflected in greater analgesic medication use. Pain upon removal of foams has been observed in other studies, and it was occasionally necessary to administer general anesthesia^2^^,^ 13. Other complications found were mild: maceration and perilesional contact dermatitis, a thread of necrosis on the edge of the wound (single case), and small hematoma (50 mL). Other papers have also associated NPWT with mild and self-limiting problems^39^. In the present study, there were no deaths or worsening of lesions. No complications had systemic repercussions or required intensive interventions.

## Conclusion

SVDM was effective in cleaning, granulating, improving clinical appearance, and optimizing the indication for surgical closure in treated wounds. However, the effectiveness was reduced in recalcitrant wounds. SVDM also proved to be safe, as it did not result in severe complications and had a favorable risk-benefit ratio for most problems encountered.
